# Bioinformatics Analysis of *MAPKKK* Family Genes in *Medicago truncatula*

**DOI:** 10.3390/genes7040013

**Published:** 2016-04-04

**Authors:** Wei Li, Hanyun Xu, Ying Liu, Lili Song, Changhong Guo, Yongjun Shu

**Affiliations:** Key Laboratory of Molecular Cytogenetic and Genetic Breeding of Heilongjiang Province, College of Life Science and Technology, Harbin Normal University, Harbin 150025, China; selva1993@163.com (W.L.); xuhanyun2016@sina.com (H.X.); 15114599330@163.com (Y.L.); sllsyf@163.com (L.S.)

**Keywords:** *Medicago truncatula*, *MAPKKK*, phylogenetic analysis, expression analysis, abiotic stresses

## Abstract

Mitogen-activated protein kinase kinase kinase (*MAPKKK*) is a component of the *MAPK* cascade pathway that plays an important role in plant growth, development, and response to abiotic stress, the functions of which have been well characterized in several plant species, such as *Arabidopsis*, rice, and maize. In this study, we performed genome-wide and systemic bioinformatics analysis of *MAPKKK* family genes in *Medicago truncatula*. In total, there were 73 *MAPKKK* family members identified by search of homologs, and they were classified into three subfamilies, *MEKK*, *ZIK*, and *RAF*. Based on the genomic duplication function, 72 *MtMAPKKK* genes were located throughout all chromosomes, but they cluster in different chromosomes. Using microarray data and high-throughput sequencing-data, we assessed their expression profiles in growth and development processes; these results provided evidence for exploring their important functions in developmental regulation, especially in the nodulation process. Furthermore, we investigated their expression in abiotic stresses by RNA-seq, which confirmed their critical roles in signal transduction and regulation processes under stress. In summary, our genome-wide, systemic characterization and expressional analysis of *MtMAPKKK* genes will provide insights that will be useful for characterizing the molecular functions of these genes in *M. truncatula.*

## 1. Introduction

MAP kinase signaling cascade pathways have been well identified and characterized in many plants [1]. These *MAPK* pathways are characterized with important roles in plant growth, development, and response to abiotic stress [2]. The *MAPK* pathway is minimally consisted of three members, a *MAPKKK* (*MAPK* kinase kinase), a *MAPKK* (*MAPK* kinase), and a *MAPK* [3,4]; they interact and transmit signals from upstream receptors to downstream function targets by phosphorylation function [5,6]. Among these *MAPK* families, the *MAPKKK* family is the largest, with more gene members than other families. They are further classified into three subfamilies, *RAF*, *MEKK*, and *ZIK*, based on characteristic sequence motifs [3].

In plants, the functions of *MAPKKK* family members are extensively studied. For instance, in *Arabidopsis*, three *MAPKKK* genes (*ANP1*, *ANP2*, and *ANP3*) regulate plant cell division [7], while another *MAPKKK* gene, *YODA*, regulates stomata development [8]. Meanwhile, some *MAPKKK* genes are reported to have a function in the signal transduction pathway’s response to various stresses [9]. For example, *CTR1* and *EDR1*, belonging to the *Arabidopsis RAF MAPKKK* subfamily, negatively regulate ethylene signaling transduction and participate in pathogen resistance [10,11,12]. Similar research has shown that DSM1, a Raf-like MAPKKK gene, improves drought tolerance through ROS scavenging in rice [13]. In the tomato, *SlMAPKKK*ɛ is reported to positively regulate cell death, functioning in plant immunity and disease resistance [14]. Recently, many plant genomes have become available; lots of *MAPKKK* genes have been identified by genome-wide searching methods, including *Arabidopsis* (80 members) [15], rice (75 members) [16], maize (74 members) [17], and soybeans (150 members) [18].

*Medicago truncatula* is an annual legume plant that can form a symbiotic association with soil bacteria called rhizobia. Because of its small, diploid genome, short life cycle, self-fertility, and high genetic transformation efficiency, *M. truncatula* has become an excellent legume model plant [19]. When its genome sequence was released, Neupane *et al.* investigated two *MAPK* families, *MAPK* and *MAPKK*, and their reports showed that *MAPK* signaling cascade pathways played important roles in tissue development, such as leaf, root, and nodule [20]. However, the largest family, *MAPKKK*, has not been identified on a genome level; their function is poorly characterized in *M. truncatula*.

In this study, we performed a genome-wide analysis of the *MAPKKK* family in *M. truncatula*, including phylogenetic analysis, chromosomal localization, and gene duplication analysis. Meanwhile, we also investigated their expression profiles by microarray data and an RNA-seq experiment, and explored their function in plant development and response to stresses. These findings would be valuable for understanding *MAPK* cascade function and promoting their utilization in legumes’ genetic improvement.

## 2. Materials and Methods

### 2.1. Identification of the MAPKKK Gene Family in Medicago truncatula

The *Medicago truncatula* genome sequences were downloaded from JCVI (http://jcvi.org/medicago/, Mt4.0) [19]. *MAPKKK* protein sequences of *Arabidopsis* were collected and used as queries to search against the *M. truncatula* genome using the BLASTP program with e-values of 1E–5. The blast hits were confirmed to contain protein kinase domain (PF00069) [21] using HMMER [22] tools. These proteins from the same gene locus were identified as protein duplications, and the redundancies were removed with the longest one kept. The remaining one was identified as a *MAPKKK* family member. All of the putative *MtMAPKKK* family genes were aligned to *Arabidopsis MAPKKK* proteins to classify them into different subfamilies as Janitza *et al.* described [23]. Meanwhile, all of the annotation information of these *MtMAPKKK* genes was retrieved from the *M. truncatula* genome website, and their structures were displayed using GSDS software [24].

### 2.2. Phylogenetic Analysis of the MtMAPKKK Genes in M. truncatula

The protein sequences of *MtMAPKKK* genes were aligned using ClustalW with the default parameters [25]. The results were used for phylogenetic analysis using MEGA (Version 4.0), and an unrooted phylogenetic tree was generated using the neighbor-joining (NJ) method with the following parameters: Poisson correction, pair-wise deletion, and 1,000 bootstrap replicates [26].

### 2.3. Chromosomal Location and Gene Duplication of MtMAPKKK Genes

Positional information about all of the *MAPKKK* genes was retrieved from the *M. truncatula* genome, and the nucleotide sequences of these genes were used as query sequences for a BLASTN search against each other to explore gene duplication, with similarities of more than 85%. In addition, duplications between the *MAPKKK* genes were also identified and complemented using the PGDD database (http://chibba.agtec.uga.edu/duplication/) [27]. Based on the space between duplication gene pairs, these duplications were classified into tandem duplications (TD, separated by four or fewer gene loci) and segmental duplications (SD, separated by more than five genes), as in our previous description [28]. The chromosome locations of *MAPKKK* genes in *M. truncatula* were drawn using the Circos software (http://circos.ca/) [29], and duplicated genes between different chromosomes or loci were also linked with colored lines in the diagrams.

### 2.4. Expression Analysis of MtMAPKKK Genes in Growth and Development

Gene expression data involving major organ systems development, particularly the development of nodules and seeds, were downloaded from the *Medicago truncatula* Gene Expression Atlas (MtGEA) Project (MtGEA, http://mtgea.noble.org/v3/) [30]. Meanwhile, genome-wide transcriptome data from *M. truncatula* in different tissues during development were downloaded from the NCBI short read archive database (SRA database) (http://www.ncbi.nlm.nih.gov, Accession numbers SRX099057–SRX099062). The expressional profiles of *MtMAPKKK* genes were retrieved from these expression data, and they were analyzed, clustered, and displayed using ggplot2 of R software (Version 3.1.0).

### 2.5. Expression Analysis of MtMAPKKK Genes’ Response to Abiotic Stress

An RNA-seq data previously reported by our group, Shu *et al.* [28], was analyzed for differential expression of *MtMAPKKK* genes involved in abiotic stress. In brief, the RNA-seq experiment was performed as follows: the seeds of *M. truncatula* (cv. Jemalong A17) were germinated and grown for eight weeks. Then, these seedlings were grown under normal conditions, cold stress (4 °C), freezing stress (−8 °C), osmotic stress (300 mM mannitol), salt stress (200 mM NaCl), and ABA (100 μM ABA). For each condition, five randomly chosen whole seedlings were pooled to form a biological replicate after three hours’ treatment. All plant samples were frozen in liquid nitrogen and stored at −80 °C until use. Total RNA was extracted from six samples, and they were sent to BGI-Shenzhen Ltd. (Shenzhen, China) for construction of pair-end cDNA libraries and performing Illumina sequencing. *MtMAPKKK* gene expressions across six treatment samples were evaluated using the TopHat [31] and Cufflinks [32] software, and they were analyzed, clustered, and displayed using the ggplot2 of R software (Version 3.1.0).

## 3. Results

### 3.1. Identification and Characterization of MAPKKK Family in M. truncatula

**Figure 1:**
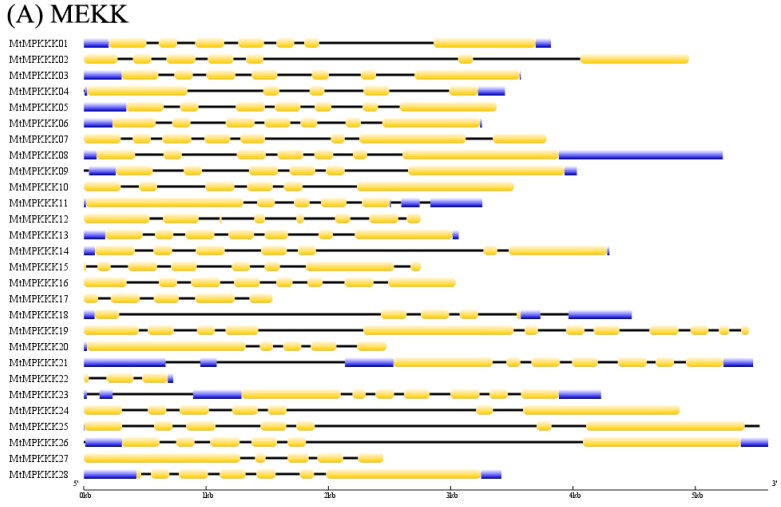

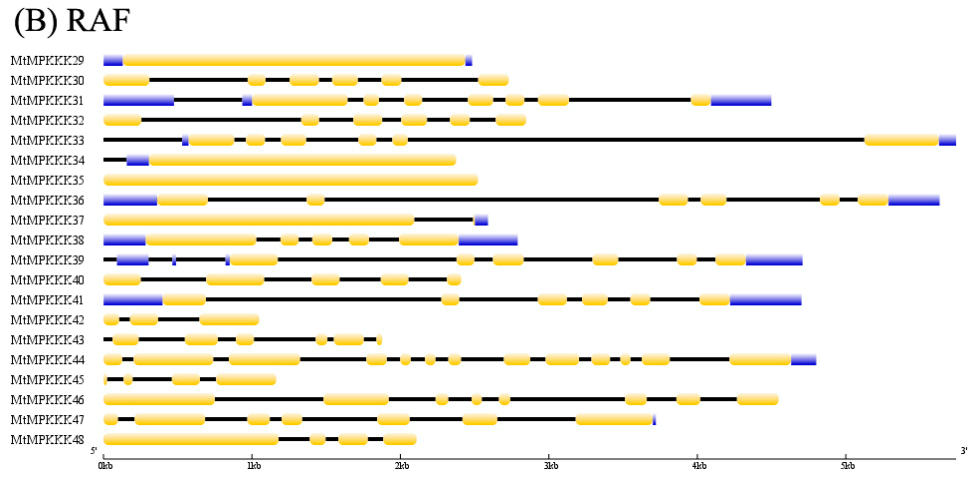

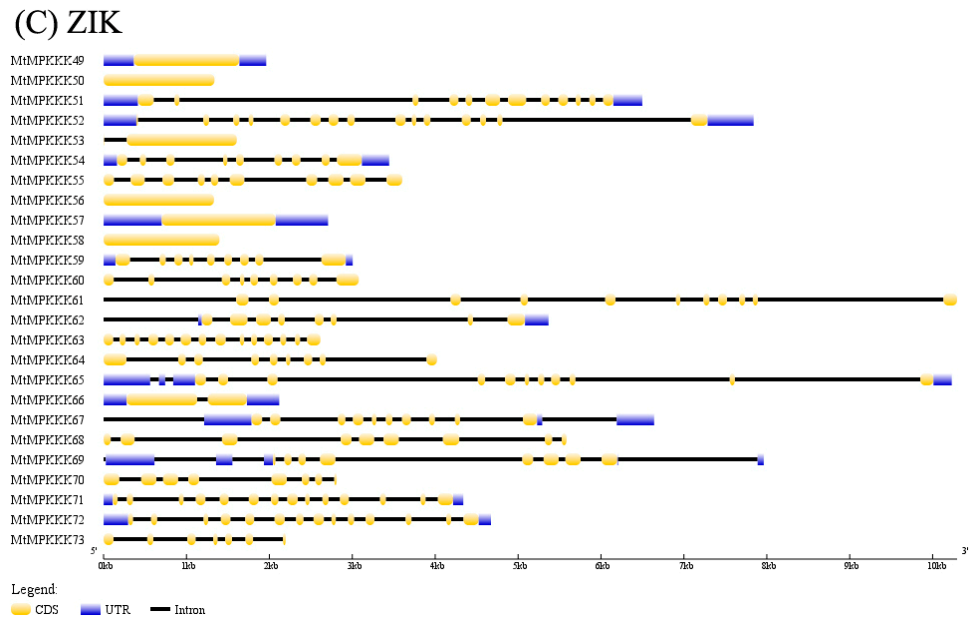
The gene structure analysis of *MAPKKK* gene family in *Medicago truncatula*. (**A**) MEKK subfamily; (**B**) RAF subfamily; (**C**) ZIK subfamily.

To identify the *MAPKKK* genes family, we used 80 *Arabidopsis MAPKKK* genes as query sequences to perform a blast search against the *M. truncatula* genome sequence. In total 73 protein sequences from the *M. truncatula* were homologous to *Arabidopsis MAPKKK* genes, with protein kinase domain (PF00069), and they were identified as *MAPKKK* family genes, named *MtMAPKKK*01–73 based on their locations on the chromosomes, as Table 1 shows. According to homology with *Arabidopsis MAPKKK*s, they were classified into three subfamilies, *MEKK* (28 members), *RAF* (20 members), and *ZIK* (25 members). The amino acid sequence lengths of *MtMAPKKK* varied from 118 (*MtMAPKKK*73) to 1107 (*MtMAPKKK*19) amino acids (aa); average length was 532 aa. The number of introns was highly divergent, from one to 14, as Figure 1 shows, which is consistent with *MAPKKK* genes in *Arabidopsis*. The introns’ distribution is subfamily specific: members of the *ZIK* subfamily generally contained the most and the longest introns; next was the *MEKK* subfamily, and last was the *RAF* subfamily, which may be involved in expression regulation [33].

### 3.2. Phylogenetic Analysis of MtMAPKKK Genes

**Figure 2:**
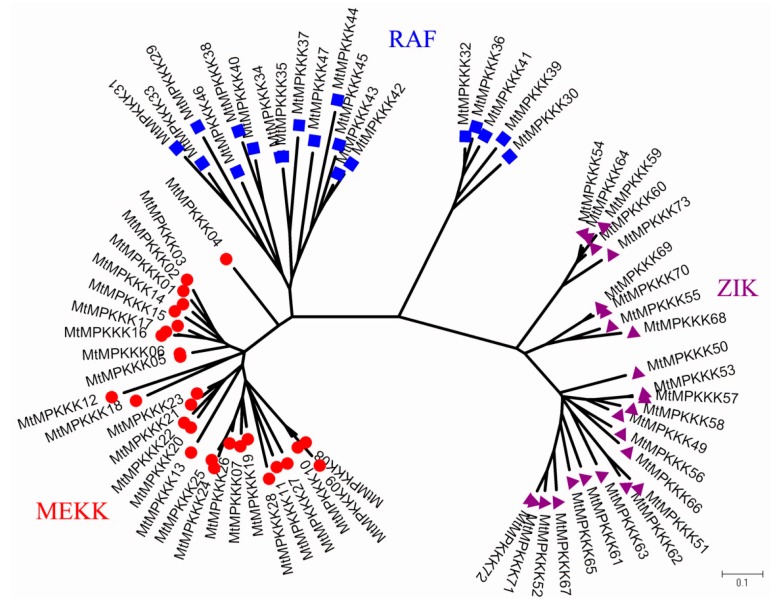
Phylogenetic tree analysis of the *MAPKKK* gene family in *Medicago truncatula*.

To investigate the evolutionary relationships of *MtMAPKKK* genes, we performed multiple sequence alignment and phylogenetic analysis. The results showed that these *MtMAPKKK* genes were clearly divided into three subfamilies (see Figures 2 and S1), which confirmed their previous classification in *Arabidopsis* homologues. As shown in Figure 2, there was only one branch in the *MEKK* subfamily, which suggested that the *MEKK* subfamily was highly conserved. On the other hand, there were two branches in the phylogenetic tree of the *RAF* subfamily and three in the *ZIK* subfamily, which implied that their functions diverged. In the three types, there were many *MtMAPKKK* genes that diverged less and clustered together; for example, *MtMAPKKK*01–03 and *MtMAPKKK*14–17 in *MEKK* subfamily. These clusters indicated that the *MtMAPKKK* genes have undergone expansion through gene duplication during the *M. truncatula* genome evolution process, and these clusters conferred a number of paralogous genes, which might perform the same function in biological processes.

### 3.3. Chromosomal Location and Duplication Analysis of MtMAPKKK Genes

**Figure 3:**
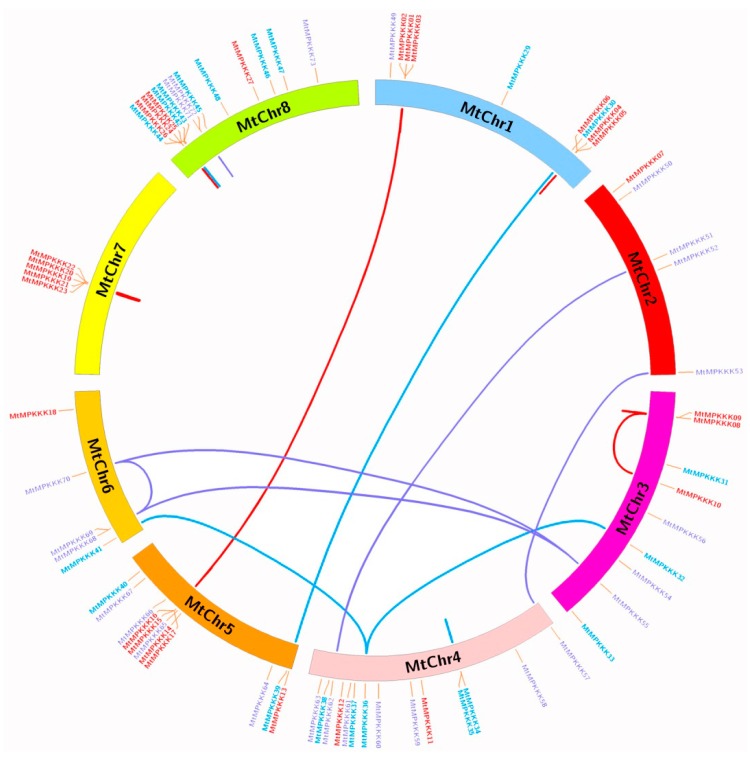
Chromosomal distribution and expansion analysis of *MtMAPKKK* genes in *Medicago truncatula.* Red lines show duplications between members of the *MEKK* subfamily, blue lines show duplications between members of the *RAF* subfamily, and purple lines show duplications between members of the *ZIK* subfamily.

Based on physical locations of the *MtMAPKKK* genes on *M. truncatula* chromosomes, they were displayed using Circos software, as Figure 3 shows. The results showed that the *MtMAPKKK* genes (except *MtMAPKKK*28) are distributed across eight chromosomes, and each chromosome holds different contents of *MtMAPKKK* genes, ranging from five to 14 members. The chromosomes MtChr4 and MtChr8 contained the most *MtMAPKKK* genes (14 members), while chromosomes MtChr7 and MtChr9 held the fewest members (five genes). In addition, by blast analysis and database search, we identified 35 pairs of gene duplication events in these *MtMAPKKK* genes, which arose from tandem duplications (22 pairs) and segment duplications (13 pairs). These duplications led to expansion of the *MtMAPKKK* family in the *M. truncatula* genome. Among these duplications, tandem duplications have resulted in *MtMAPKKK* gene clusters or hot regions; for instance, *MtMAPKKK*19-22 results in an *MEKK* subfamily cluster in MtChr7. The segment duplication has resulted in *MAPKKK* members in different chromosomes—for example, duplication between *MtMAPKKK*01–03 and *MtMAPKKK*14–17 had expanded the *MEKK* subfamily from MtChr1 to MtChr5; the clustering was also confirmed by phylogenetic analysis.

### 3.4. In Silico Expression Analysis of MtMAPKKK Genes Involved in Growth and Development

**Figure 4:**
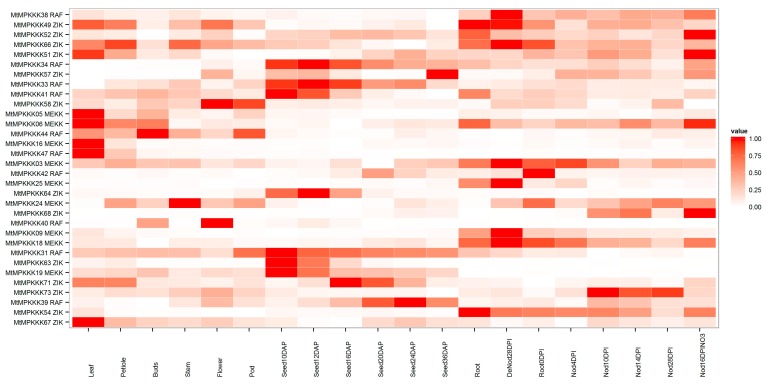
Expression profile cluster analysis of *MtMAPKKK* genes involved in growth and development. The expression data were clustered and displayed using software ggplot2. The DAP infers days after pollination, while DPI infers days after inoculation; the information on samples is from Benedito *et al.* [30].

**Figure 5:**
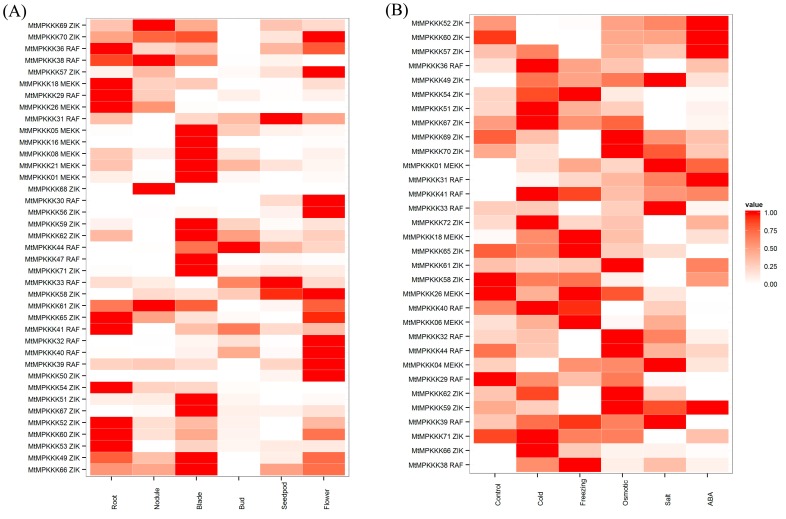
Expression profile cluster analysis of *MtMAPKKK* genes involving in tissue development (**A**) and response to abiotic stress (**B**).

To investigate *MtMAPKKK* genes’ expression in plant growth and development, expression data of *M. truncatula* were collected from MtGEA and NCBI, including microarray data and high-throughput sequencing data. Expression profiles of *MtMAPKKK* genes were retrieved and analyzed, as Figure 4 and Figure 5 show. According to the microarray data, these *MtMAPKKK* genes were clustered into three groups. Group A genes, including *MtMAPKKK*05, 06, 16, 44, 47, 49, 51, 66, and 67, were expressed in different tissues; expression was associated with tissue development. Group B genes, including *MtMAPKKK*19, 31, 33, 34, 41, 57, 63, 64, and 71, were expressed during seed development. In the last group, including *MtMAPKKK*03, 06, 09, 18, 24, 25, 38, 42, 49, 52, 54, 66, 68, and 71, expression was associated with the nodulation process in *M. truncatula*. These results suggested that *MtMAPKKK* genes were expressed in specific tissues or during different stages of development, with a potential role in the development processes of *M. truncatula*. In addition, expression profiles of these *MtMAPKKK* genes from high-throughput sequencing were confirmed by the results in microarray expression (see Figure 5A); for example, *MtMAPKKK*40, 49, and 58 were specifically expressed in flowers both in microarray and high-throughput sequencing. Similarly, *MAPKKK*44 was also confirmed to be expressed in buds by two datasets. However, there were 39 *MtMAPKKK* genes detected in high-throughput sequencing data, which was slightly more than in the microarray dataset (32 *MtMAPKKK* genes).

### 3.5. Expression Analysis of MtMAPKKK Genes in Response to Abiotic Stresses

With the development of next sequencing technology, we have performed RNA-seq to identified *MtMAPKKK* genes’ response to abiotic stress. There were 32 *MtMAPKKK* genes expressed in six samples, while 15 *MtMAPKKK* genes were differentially expressed under abiotic stress, including cold (five members), freezing (five members), osmotic (two members), salt (eight members) and ABA (four members) treatments (see Figure 5B). Among these *MtMAPKKK* genes, *MtMAPKKK*49 was upregulated by all stresses, while other *MtMAPKKK* genes were differentially affected by treatment. For example, *MtMAPKKK*36, 41, 51, 54, 66, 67, 71, and 72 were highly expressed under cold stress, but had no expression or very low expression in other stresses. Similarly, *MtMAPKKK*18, 65, 26, 46, 06, and 38 were specifically expressed under freezing stress; *MtMAPKKK*32, 44, 59, 62, 69, and 70 were expressed under osmotic stress; *MtMAPKKK*01, 04, 33, 39 and 49 were present in salt stress; and *MtMAPKKK*31, 52, 57, 59, and 60 were responsive to ABA stimuli. Compared to ABA treatment, we found that there were some correlation between osmotic and salt stress with ABA treatment, such as in *MtMAPKKK*52, 57, 59, and 60. However, there were no common *MAPKKK* members expressed in both cold and freezing conditions with ABA treatment, implying that these *MtMAPKKK* genes may have a role in osmotic and salt stresses through the ABA regulation pathway, but may not have a role in cold and freezing stresses.

## 4. Discussion

In previous studies, *MAPK* signaling cascade pathways played important roles in various processes, including developmental processes, biotic, and abiotic stress responses [1,8]. To date, a large number of *MAPKKK* genes have been identified and characterized in plants, including *Arabidopsis* [14], rice [12], grapevines [34], maize [16], soybeans [17], and tomatoes [35]. However, the *M. truncatula* genome has been reported [19], and both the *MAPK* and *MAPKK* gene families have been identified and characterized [20]; until now the *MAPKKK* gene family has not been reported. In the present research, we performed genome-wide analysis for the *MAPKKK* family in *M. truncatula*, and 73 *MtMAPKKK* genes were identified by homologous searching and domain analysis. The number of *MtMAPKKK* genes is similar to their members in *Arabidopsis*, rice, maize, and tomatoes, but half that of the soybean *MAPKKK* family, which is an allopolyploid species (see Table 2). The results showed that the *MAPKKK* family is highly conserved in the plants. Based on characteristic sequence motifs, they were divided into three subfamilies, *MEKK*, *RAF*, and *ZIK*, as is done for other plants [14,12]. However, the numbers of subfamilies diverge from other plants, such as *Arabidopsis*, rice, maize, and tomatoes. The *MEKK* subfamily has 28 members, which is consistent with other plants (see Table 2), implying that it is the most conservative subfamily in *M. truncatula*. By gene duplication analysis, we identified 24 duplication events in the *MEKK* subfamily; 19 of them are tandem duplication events, which have conferred *MEKK* gene clusters in the *M. truncatula* genome, such as *MtMAPKKK*01-03, *MtMAPKKK*04-06 on MtChr01, *MtMAPKKK*14-17 on MtChr05, and *MtMAPKKK*19-23 on MtChr07. These results strongly suggested that tandem duplications mainly contributed to conservation of the *MEKK* subfamily in *M. truncatula* (see Figure 2). Compared to the *MEKK* subfamily, the *RAF* and *ZIK* subfamilies are more divergent. There are *RAF* genes in *M. truncatula*, but fewer than in other plants (see Table 2). However, the *ZIK* subfamily has more members (25), implying that *ZIK* genes have expanded. This divergence is present not in terms of numbers of members, but in gene structures. The *MEKK* genes contain 4 to 7 introns, while *RAF* has 0–6 introns, and the *ZIK* subfamily varied from 0 to 13 (see Table 1 and Figure 1). The intron is an important regulator of gene expression in eukaryotes; more introns generally indicate more complex regulation, which may be an important role in complex biological processes, such as tissue development in response to abiotic stress [33]. It is worth noting that *MtMAPKKK* gene duplication conferred similar gene structures, such as *MtMAPKKK*01::03 (*MEKK*), *MtMAPKKK*05::06 (*MEKK*), *MtMAPKKK*36::41 (*RAF*), *MtMAPKKK*71::72 (*ZIK*), *etc.*, which suggests they would have similar expression profiles and function in *M. truncatula*.

**Figure 6:**
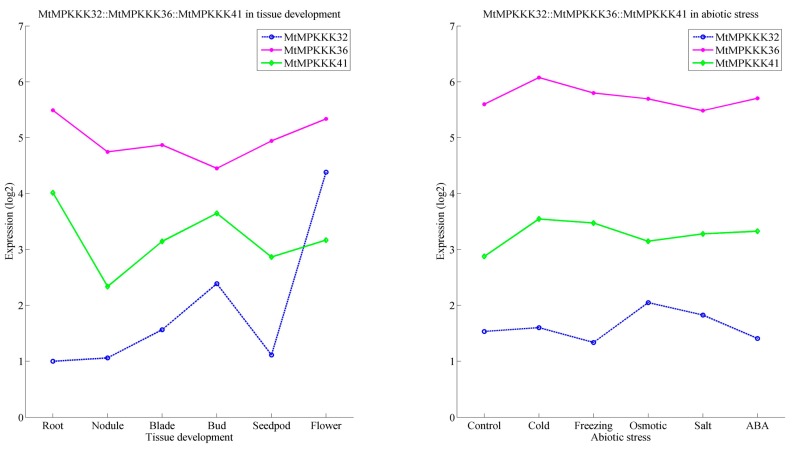
The expression profiles of *MtMAPKKK32::36::41* involved in tissue development and response to abiotic stress.

In *Arabidopsis*, the *MAPKKK* family has undergone a large expansion through gene duplication, resulting in 80 members across three subfamilies [36]. In the expansion process, there were a number of paralogous genes produced by gene duplication, whose expressions and functions differed in the evolutionary process [37]. In *M. truncatula*, *MtMAPKKK* genes have also undergone a gene duplication process, which conferred paralogous gene pairs, such as *MtMAPKKK*01::03 (*MEKK*), *MtMAPKKK*05::06 (*MEKK*), *MtMAPKKK*32::36::41 (*RAF*), *MtMAPKKK*71::72 (*ZIK*), as previously described. Generally, these genes have similar expression profiles, such as *MtMAPKKK*05::06 (*MEKK*). These two *MEKK* genes were both highly expressed in leaves, while they were absent or less expressed in the seed development process (see Figure 4). A similar process happened in *MtMAPKKK*71::72 (*ZIK*): they were both highly induced by cold stress, but less expressed in response to other stresses (see Figure 5). However, the duplication gene pairs can also differ in expression level, for example, *MtMAPKKK*32::36::41. They were expanded to three chromosomes (MtChr03, 04, and 06; see Figure 3) by segment duplication events, and they had different expression levels (see Figure 6). *MtMAPKKK*36 was more highly expressed than *MtMAPKKK*41, and *MtMAPKKK*41 was more highly expressed than *MtMAPKKK*32. In addition, *MtMAPKKK*36 and 41 expression constituted the tissue development and response to abiotic stresses, implying their identical and essential functions during plant development and abiotic stress response processes. By contrast, *MtMAPKKK*32 was expressed in tissue development, specifically in flowers, which indicated its important role in flower development (see Figure 6).

On a global scale, plant growth and development are threatened by various abiotic stresses, such as cold, drought, and salinity [38]. Therefore, plants should have the ability to constantly adapt to unfavorable environmental conditions. They employ complex regulatory mechanisms for undergoing physiological and biochemical changes in response to stresses [8,39]. *MAPK* signaling cascade pathways play a remarkably important role in the sensing and transmitting of stress signals, which is an essential step in the establishment of tolerance to various stresses [1,10]. The *MAPKKK* family has the most members in *MAPK* signaling pathways, and they are widely expressed to regulate plant processes, including growth, development, and response to abiotic stresses [6]. In our study, we investigated the expression profiles of *MtMAPKKK* genes using microarray and RNA-seq data from NCBI and our previous research [28]. The expression data were clustered and visualized using a heat-map method, and the results show a wide range of expression levels and distinct regulation during plant development and response to biotic and/or abiotic stresses. In *M. truncatula* growth and tissue development, the expression of *MtMAPKKK* genes overlaps among these tissues and organs. It is notable that many *MtMAPKKK* genes are typically expressed in specific tissues, such as *MtMAPKKK*05, 06, and 16 (*MEKK* subfamily) in leaves (see Figure 4); for *MtMAPKKK*40, 41, present in flowers, this finding is consistent with how *MAPKKK* genes are expressed in *Arabidopsis* [38]. Based on our analysis of abiotic stress expression data, we found that a large number of *MtMAPKKK* genes were highly responsive to abiotic stress; some of them were specifically responsive to selected abiotic stresses (see Figure 5). For example, *MtMAPKKK*66, 71, and 72 (*ZIK* subfamily) were induced by cold stress; *MtMAPKKK*33 was induced by salt stress; *MtMAPKKK*69, 70 were repressed by freezing stress. However, Menges *et al.* have investigated the expressions of all members of the *MAPKKK* family through a large number of microarrays in *Arabidopsis* [37]; because of limited samples, there are still 38 *MtMAPKKK* genes that have not been implicated in *M. truncatula* development and/or response to abiotic stress in the present research. We need more biological samples involving various development processes, or more time points of abiotic stress, to reveal the expression profiles of *MtMAPKKK* genes, which will be useful for determining their function in the future.

## 5. Conclusions

In summary, we have identified 73 *MtMAPKKK* genes in *M. truncatula*; they were classified into three subfamilies based on phylogenetic analysis. Meanwhile, their expression profiles have been investigated using microarray and a high-throughput sequencing dataset; the results revealed their regulation roles in plant growth and tissue development, especially their essential functions in nodule development. In addition, an RNA-seq experiment was performed to explore their regulation in response to abiotic stresses, implying that *MAPKKK* family genes broadly participated in the abiotic response process in *M. truncatula*. The information from this investigation will be useful for the identification and characterization of *MtMAPKKK* genes whose function will be explored in the future.

## Figures and Tables

**Table 1:** List of all *MtMAPKKK* genes identified in the *Medicago truncatula* genome.

Gene Name	Gene Locus	Chromosome Location	AA	Introns	Family Group
MtMPKKK01	Medtr1g021610	chr1:6496817-6500636	671	6	*MEKK*
MtMPKKK02	Medtr1g021630	chr1:6502386-6507333	677	6	*MEKK*
MtMPKKK03	Medtr1g021635	chr1:6514070-6517643	675	6	*MEKK*
MtMPKKK04	Medtr1g105615	chr1:47604242-47607699	507	4	*MEKK*
MtMPKKK05	Medtr1g105800	chr1:47742832-47746207	654	6	*MEKK*
MtMPKKK06	Medtr1g105820	chr1:47757298-47760553	672	6	*MEKK*
MtMPKKK07	Medtr2g011280	chr2:2723023-2726806	842	7	*MEKK*
MtMPKKK08	Medtr3g019420	chr3:5452378-5457705	822	6	*MEKK*
MtMPKKK09	Medtr3g019530	chr3:5500322-5504353	779	5	*MEKK*
MtMPKKK10	Medtr3g051420	chr3:20341370-20344887	781	5	*MEKK*
MtMPKKK11	Medtr4g081730	chr4:31738243-31741501	667	6	*MEKK*
MtMPKKK12	Medtr4g117800	chr4:48882742-48885495	492	7	*MEKK*
MtMPKKK13	Medtr5g005520	chr5:482150-485215	671	6	*MEKK*
MtMPKKK14	Medtr5g065130	chr5:27367690-27371987	666	6	*MEKK*
MtMPKKK15	Medtr5g068250	chr5:28854252-28857008	554	7	*MEKK*
MtMPKKK16	Medtr5g068260	chr5:28859095-28862135	667	7	*MEKK*
MtMPKKK17	Medtr5g068290	chr5:28874322-28875866	359	4	*MEKK*
MtMPKKK18	Medtr6g083980	chr6:31351315-31355795	276	5	*MEKK*
MtMPKKK19	Medtr7g056420	chr7:19934346-19939785	1107	11	*MEKK*
MtMPKKK20	Medtr7g056617	chr7:20229172-20231645	668	4	*MEKK*
MtMPKKK21	Medtr7g056647	chr7:20269305-20274777	688	8	*MEKK*
MtMPKKK22	Medtr7g056657	chr7:20284636-20285367	157	2	*MEKK*
MtMPKKK23	Medtr7g056680	chr7:20300556-20304825	660	8	*MEKK*
MtMPKKK24	Medtr8g013560	chr8:4119594-4124466	829	6	*MEKK*
MtMPKKK25	Medtr8g013580	chr8:4129624-4135154	830	7	*MEKK*
MtMPKKK26	Medtr8g013620	chr8:4158808-4164403	785	5	*MEKK*
MtMPKKK27	Medtr8g465580	chr8:23390859-23393309	653	4	*MEKK*
MtMPKKK28	Medtr0090s0020	scaffold0090:7455-10869	727	6	*MEKK*
MtMPKKK29	Medtr1g064560	chr1:28404485-28406967	769	0	*RAF*
MtMPKKK30	Medtr1g103270	chr1:46733948-46736677	383	5	*RAF*
MtMPKKK31	Medtr3g047890	chr3:15980547-15985045	506	7	*RAF*
MtMPKKK32	Medtr3g078110	chr3:35189304-35192151	364	5	*RAF*
MtMPKKK33	Medtr3g116590	chr3:54529585-54535327	447	5	*RAF*
MtMPKKK34	Medtr4g061833	chr4:22900571-22904005	690	0	*RAF*
MtMPKKK35	Medtr4g061930	chr4:22952545-22955067	841	0	*RAF*
MtMPKKK36	Medtr4g106980	chr4:43970220-43975851	393	5	*RAF*
MtMPKKK37	Medtr4g111925	chr4:46269757-46272346	702	1	*RAF*
MtMPKKK38	Medtr4g125260	chr4:51937267-51940056	515	4	*RAF*
MtMPKKK39	Medtr5g006560	chr5:981991-986717	391	7	*RAF*
MtMPKKK40	Medtr5g092120	chr5:40224346-40226755	373	4	*RAF*
MtMPKKK41	Medtr6g007603	chr6:1663170-1667871	376	5	*RAF*
MtMPKKK42	Medtr8g014740	chr8:4708849-4709897	233	2	*RAF*
MtMPKKK43	Medtr8g014860	chr8:4751582-4753459	283	5	*RAF*
MtMPKKK44	Medtr8g015340	chr8:4995169-4999969	908	12	*RAF*
MtMPKKK45	Medtr8g028115	chr8:10454560-10455723	228	3	*RAF*
MtMPKKK46	Medtr8g064690	chr8:27154367-27158914	673	7	*RAF*
MtMPKKK47	Medtr8g070910	chr8:30050035-30053755	614	6	*RAF*
MtMPKKK48	Medtr8g442290	chr8:15952474-15954582	571	3	*RAF*
MtMPKKK49	Medtr1g013700	chr1:3292908-3294869	424	0	*ZIK*
MtMPKKK50	Medtr2g016340	chr2:4982022-4983359	446	0	*ZIK*
MtMPKKK51	Medtr2g045470	chr2:19958506-19965003	466	11	*ZIK*
MtMPKKK52	Medtr2g049790	chr2:22365714-22373554	461	14	*ZIK*
MtMPKKK53	Medtr2g105010	chr2:45267272-45268878	445	1	*ZIK*
MtMPKKK54	Medtr3g086940	chr3:39410214-39413658	348	8	*ZIK*
MtMPKKK55	Medtr3g099920	chr3:45863019-45866621	493	9	*ZIK*
MtMPKKK56	Medtr3g466480	chr3:27311509-27312840	444	0	*ZIK*
MtMPKKK57	Medtr4g006970	chr4:879328-882035	458	0	*ZIK*
MtMPKKK58	Medtr4g029020	chr4:9994106-9995503	466	0	*ZIK*
MtMPKKK59	Medtr4g086855	chr4:34076831-34079835	364	8	*ZIK*
MtMPKKK60	Medtr4g099240	chr4:41124392-41127469	338	8	*ZIK*
MtMPKKK61	Medtr4g114670	chr4:47183905-47194199	382	10	*ZIK*
MtMPKKK62	Medtr4g123940	chr4:51095933-51101333	352	7	*ZIK*
MtMPKKK63	Medtr4g128820	chr4:53613425-53616040	453	13	*ZIK*
MtMPKKK64	Medtr5g013550	chr5:4318757-4322778	339	8	*ZIK*
MtMPKKK65	Medtr5g067150	chr5:28353988-28364218	374	12	*ZIK*
MtMPKKK66	Medtr5g075100	chr5:31902979-31905097	441	1	*ZIK*
MtMPKKK67	Medtr5g088350	chr5:38344231-38350871	339	10	*ZIK*
MtMPKKK68	Medtr6g012980	chr6:4042934-4048518	432	8	*ZIK*
MtMPKKK69	Medtr6g012990	chr6:4049178-4057281	362	10	*ZIK*
MtMPKKK70	Medtr6g048250	chr6:17403678-17408073	360	7	*ZIK*
MtMPKKK71	Medtr8g024590	chr8:9075528-9079866	436	13	*ZIK*
MtMPKKK72	Medtr8g024600	chr8:9083996-9088668	437	13	*ZIK*
MtMPKKK73	Medtr8g088740	chr8:36861632-36863829	188	6	*ZIK*

**Table 2:** The numbers of *MAPKKK* genes in plant genomes.

Species	*MAPKKK*			Total number of *MAPKKK*s
	*MEKK*	*RAF*	*ZIK*	
*Arabidopsis*	21	48	11	80
tomato	33	40	16	89
rice	22	43	10	75
maize	22	46	6	74
soybean	34	92	24	150
*M. truncatula*	28	20	25	73

## Supplementary Materials

The following figure are available online at www.mdpi.com/2073-4425/7/4/13/s1. Figure S1: Phylogenetic tree analysis of both *MtMAPKKK* and *AtMAPKKK* genes.

## Abbreviations

The following abbreviations are used in this manuscript:

*MAPKKK*: Mitogen-activated protein kinase kinase kinase
MtGEA: *Medicago truncatula* Gene Expression Atlas
NCBI: National Center for Biotechnology Information
ROS: reactive oxygen species
SRA: short read archive
MtChr: *M. truncatula* chromosomes
