# Supplementary material for: Bioinformatics Analysis of MAPKKK Family Genes in Medicago truncatula
Source: Genes (Basel). 2016 Apr 4;7(4):13. doi: 10.3390/genes7040013 (PMC4846843; doi:10.3390/genes7040013)
Supplement: Supplementary file 1 [file genes-07-00013-s001.pdf]

Supplementary Material

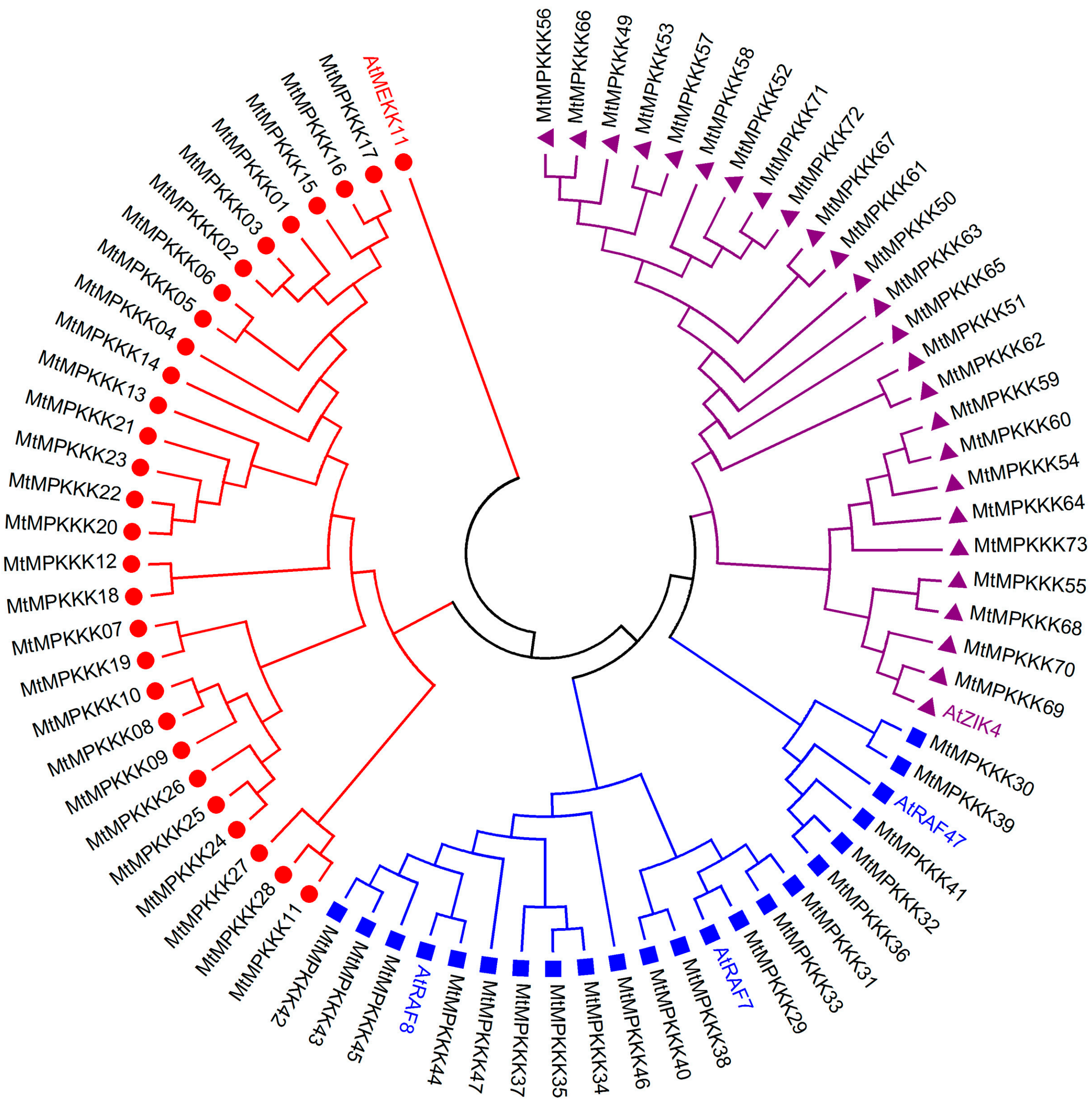

Figure S1. Phylogenetic tree analysis of both *MtMAPKKK* and *AtMAPKKK* genes.
